# Supplementary material for: Anxiety and depression among medical doctors in Catalonia, Italy, and the UK during the COVID-19 pandemic
Source: PLoS One. 2021 Nov 2;16(11):e0259213. doi: 10.1371/journal.pone.0259213 (PMC8562811; doi:10.1371/journal.pone.0259213)

Supplementary Online Content For:

Anxiety and Depression among Medical Doctors in Catalonia, Italy, and the UK during the COVID-19 Pandemic

Climent Quintana-Domeque; Ines Lee; Anwen Zhang; Eugenio Proto;

Michele Battisti; Antonia Ho

This supplemental material has been provided by the authors to give readers additional information about their work.

This document contains:

Additional Tables 2
Table S1: Prevalence of and mortality from COVID-19 2
Table S2: Survey details following AAPOR survey disclosure guidelines 3
Table S3: Place of work among UK respondents 4
Table S4. Original and final sample sizes 5
Table S5: Response rates by institutions 6
Table S6: Definition of key variables 7
Table S7: Logit odds-ratios and 95% confidence intervals 8
Additional Figures 9
Figure S1: Example email invitation June 2020 (COMB) 9
Figure S2: Example email invitation June 2020 (RCPSG) 10
Figure S3: Example email invitation November 2020 (RCSEd) 11
Figure S4: Prevalence of anxiety and depression symptoms by intensity 12

Additional Tables

Table S1: Prevalence of and mortality from COVID-19

	Catalonia		Italy		UK	
	Round 1	Round 2	Round 1	Round 2	Round 1	Round 2
COVID-19 cases per 100,000	33.3	809.6	12.5	836.3	55.6	927.1
COVID-19 deaths per 100,000	2.4	22.2	2.2	30.7	4.4	17.8

Note: Percentages reported in parentheses. Data on COVID-19 cases and deaths comes from idescat.cat (Catalonia), ourworldindata.org (Italy), and GOV.UK (UK). Round 1 corresponds to June 2020 for all countries; Round 2 corresponds to November 2020 for Catalonia and the UK and December 2020 for Italy.

Table S2: Survey details following AAPOR survey disclosure guidelines

BASIC DISCLOSURE ELEMENTS	DETAILS
Survey sponsor	Surveys were distributed online by the medical organizations (COMB, COMG, ANAAO-ASSOMED, FIMMG, RCSED, RCPSG) via their mailing lists.
Survey/Data collection supplier	Researchers from the universities of Cambridge, Exeter and Glasgow designed the survey in Qualtrics.
Population represented	Medical doctors in Catalonia, Italy and the UK.
Sample size	5,275
Mode of data collection	Online
Type of sample (probability/non-probability)	Non-probability (COMG, ANAAO-ASSOMED, FIMMG, RCSED, RCPS) and probability (COMB).
Start and end dates of data collection	May 29, 2020 to June 30, 2020.<br>November 1, 2020 to December 31, 2020.
Margin of sampling error for total sample	NA
Margin of sampling error for key subgroups	NA
Are the data weighted?	The data are not weighted. Weights are not suitable in our context because the available weights reflect the composition of the respective institutions regardless of the characteristics of their members, while our sample focuses on a subsample of the underlying population (see Table S4).
Contact for more information	Climent Quintana-Domeque, PhD<br>University of Exeter, Business School, Department of Economics<br>c.quintana-domeque@exeter.ac.uk

Note: The code and data are deposited in the Harvard Dataverse repository: https://doi.org/10.7910/DVN/DRSMYH.

Table S3: Place of work among UK respondents

	RCPSG Round 1	RCPSG Round 2	RCSEd Round 1	RCSEd Round 2
England	35.5%	41.4%	74.7%	71%
Northern Ireland	2.2%	4.5%	4.3%	7.1%
Scotland	61.5%	52.2%	16%	18.6%
Wales	0.8%	1.9%	5%	3.3%

Note: Round 1 corresponds to June 2020; round 2 corresponds to November 2020.

Table S4. Original and final sample sizes

	Catalonia		Italy		UK	
	COMB	COMG	Anaao-Assomed	FIMMG	RCPSG	RCSEd
**Round 1**						
Period	June 2020	June 2020	June 2020	June 2020	June 2020	June 2020
N (initial)[a]	1,067	275	1,524	1,136	399	575
N (excluding missing info)[b]	886	213	1,223	858	333	461
N (excluding duplicates)[c]	866	207	1,190	840	328	451
N (excluding different region)[d]	828	195	1,121	794	261	321
N (excluding other occupation)[e]	742	174	873	780	245	292
N (excluding other cases)[f]	706	170	862	775	231	281
**Round 2**						
Period	Nov 2020	Nov 2020	Dec 2020	Dec 2020	Nov 2020	Nov 2020
N (initial)[a]	1,023	432	1,021	460	228	404
N (excluding missing info)[b]	835	356	856	360	175	238
N (excluding duplicates)[c]	824	346	842	347	174	237
N (excluding different region)[d]	781	324	802	328	162	217
N (excluding other occupation)[e]	704	299	632	327	160	189
N (excluding other cases)[f]	688	285	614	323	157	183

[a] Raw data.

[b] Excluding missing information on sex, age, household composition, occupation and specialty.

[c] Excluding duplicates. Duplicates are identified as observations with the same IP address, sex, age, number of children below 5 years in the household, number of children 6-17 years in the household, number of adults 18-59 years in the household, number of adults 60 years and above in the household, occupation and specialty.

[d] Excluding respondents working in a different region/country (e.g. respondents in COMB working outside Catalonia, respondents in RCSEd working outside the UK).

[e] Excluding other occupations (e.g. biologists).

[f] Excluding other cases (e.g. retired, on leave, shielding).

Table S5: Response rates by institutions

Institution	June 2020	November/December 2020
COMB (Catalonia)	706/5,062[a]	688/5,062[a]
COMG (Catalonia)	170/3,120	285/3,120
Anaao-Assomed (Italy)	862/23,379[b]	614/23,379[b]
FIMMG (Italy)	775/17,687	323/17,687
RCPSG (Scotland)	231/3,990	157/4,300
RCSEd (Scotland)	281/4,992	183/4,912

Note: COMB (Barcelona Medical Council) and COMG (Girona Medical Council) are medical councils; Anaao-Assomed (Union of physicians and healthcare executives) and FIMMG (Union of general practitioners) are medical unions; RCSEd (Royal College of Surgeons of Edinburgh) and RCPSG (Royal College of Physicians and Surgeons of Glasgow) are private medical associations.

[a] Of the 36,339 COMB members, we focused on 25,425 members who were under 70 years, available to be contacted via e-mail, and willing to be contacted. Within this group, COMB invited 5,062 members in June and November 2020 (19.9%).

[b] This includes a large number of members who are not medical doctors.

Note that computing the response rate is problematic for several reasons, including the following: (1) membership changes over time (especially in private associations); (2) in Anaao-Assomed and UK, members are not just medical doctors.

Table S6: Definition of key variables

Variable	Definition
**Key outcome variables**	
Anxiety	=1 if Generalized Anxiety Disorder Assessment (GAD-7) ≥ 10, =0 otherwise
Depression	=1 if Patient Health Questionnaire-9 (PHQ-9) ≥10, =0 otherwise
**Demographic and survey information**	
Woman	=1 if woman; =0 if man
Age < 60	= 1 if below 60; = 0 otherwise
Survey round 2	= 1 if surveyed in November/December 2020; = 0 if surveyed in June 2020
**Perceptions of workplace safety**	
Does not have necessary PPE	=1 if do not agree with the statement "My workplace is providing me with the necessary Protective Personal Equipment"; = 0 otherwise
Little concern for safety	= 1 if strongly agree or somewhat agree to the statement "My workplace has shown little concern for my safety"; = 0 otherwise
Feel vulnerable/exposed	= 1 if strongly agree or somewhat agree to the statement "I feel vulnerable and exposed at work"; = 0 otherwise
**Exposure to COVID-19**	
Had COVID-19 symptoms	= 1 if had COVID-19 symptoms; 0 if no COVID-19 symptoms
Directly treat COVID-19 patients	= 1 if respondent replies "yes" to the question "In the last week, did you directly look after COVID-19 patients?"; = 0 if respondent replies "no".
Help w/ COVID-19 related tasks	= 1 if respondent replies "yes" to the question "In the last week, have you been asked to help out with work related COVID-19 patients without treating them directly?"; = 0 if respondent replies "no".
≥1 COVID-19 death in workplace	= 1 if respondents says that there are positive number of doctor, nurse, or other personnel deaths in response to the question "Are you aware of any COVID-19 deaths among healthcare workers in your workplace?" ; = 0 otherwise.
**Health, health behaviors, and lifestyle**	
Normal/below-normal health	=1 if respondent rates their general health as 3 or below on a 1-5 Likert scale, where high values correspond to better health; =0 otherwise.
Underlying health condition	= 1 if respondent has underlying health conditions; = 0 otherwise
Worked ≥ 40 hours	= 1 if respondent worked 40 hours or more in the past week; = 0 otherwise
Smokes	= 1 if respondent smokes; = 0 otherwise
Had flu vaccine this season	= 1 if had flu vaccine this season; = 0 otherwise
**Household composition**	
Lives with child under 5	= 1 if lives with a child under 5; = 0 otherwise
Lives with someone over 60	= 1 if lives with someone 60 or above; = 0 otherwise
**Other indicators**	
Occupational indicators	7 occupations indicators (e.g. in the UK: Consultant, SAS doctor, Specialty registrar, Junior doctor core training, Junior doctor foundation year, General practitioner, General practitioner trainee), that = 1 if respondent's occupation equals that category; = 0 otherwise.
Institutional indicators	6 institutional indicators (COMB, COMG, Anaao-Assomed, FIMMG, RCPSG, RCSEd) that = 1 if respondent belongs to that institution; = 0 otherwise.

Table S7: Logit odds-ratios and 95% confidence intervals

	All		Catalonia		Italy		UK	
	Anxiety	Depression	Anxiety	Depression	Anxiety	Depression	Anxiety	Depression
**Demographics**								
Woman	1.77***	1.76***	1.51*	1.45*	1.92***	2.00***	1.63*	1.81**
	[1.50,2.07]	[1.49,2.09]	[1.09,2.11]	[1.05,2.01]	[1.56,2.36]	[1.59,2.52]	[1.05,2.52]	[1.18,2.77]
Age<60	1.49***	1.58***	2.19***	2.16***	1.29*	1.25	2.27*	3.96**
	[1.22,1.82]	[1.28,1.96]	[1.40,3.43]	[1.39,3.35]	[1.02,1.65]	[0.96,1.64]	[1.06,4.88]	[1.68,9.30]
Survey round 2	1.01	0.9	0.78	0.84	1.04	0.81	1.51	1.43
	[0.86,1.18]	[0.76,1.06]	[0.59,1.04]	[0.63,1.10]	[0.83,1.30]	[0.63,1.05]	[0.95,2.38]	[0.91,2.23]
**Perceptions of workplace safety**								
Does not have necessary PPE	1.39***	1.27*	1.43*	1.23	1.37*	1.26	1.15	1.42
	[1.15,1.68]	[1.04,1.56]	[1.01,2.02]	[0.87,1.72]	[1.07,1.77]	[0.95,1.66]	[0.61,2.17]	[0.77,2.61]
Feel vulnerable/exposed	1.68***	1.72***	1.45*	1.72***	1.77***	1.61***	1.98**	2.40***
	[1.41,2.00]	[1.43,2.06]	[1.05,2.01]	[1.26,2.35]	[1.39,2.24]	[1.23,2.09]	[1.19,3.28]	[1.47,3.91]
Little concern for safety	1.29*	1.36**	1.35	1.43	1.25	1.52**	1.64	1.04
	[1.06,1.57]	[1.11,1.67]	[0.92,1.98]	[0.98,2.08]	[0.97,1.60]	[1.15,2.00]	[0.88,3.06]	[0.55,1.96]
**Exposure to COVID-19**								
Had COVID-19 symptoms	1.16	1.54***	1.31	1.93***	1.1	1.32	0.96	1.43
	[0.97,1.39]	[1.29,1.84]	[0.98,1.76]	[1.46,2.55]	[0.84,1.44]	[0.99,1.76]	[0.59,1.55]	[0.91,2.25]
Directly treat	1.32**	1.32**	1.39*	1.55**	1.29*	1.31*	1.34	1.12
COVID-19 patients	[1.12,1.56]	[1.11,1.57]	[1.01,1.90]	[1.14,2.10]	[1.03,1.61]	[1.03,1.68]	[0.83,2.15]	[0.70,1.78]
Help w/ COVID-19	1.20*	1.20*	1.21	0.97	1.28*	1.51**	0.98	1.03
related tasks	[1.02,1.42]	[1.01,1.43]	[0.89,1.64]	[0.72,1.31]	[1.02,1.60]	[1.18,1.92]	[0.61,1.57]	[0.65,1.62]
≥1 COVID-19 death	1.20*	1.15	1.23	1.36	1.17	1.09	1.29	1.1
in workplace	[1.02,1.41]	[0.97,1.37]	[0.88,1.73]	[0.98,1.88]	[0.95,1.44]	[0.86,1.38]	[0.83,1.98]	[0.73,1.68]
**Health status and behaviors**								
Normal/below-normal	2.58***	3.35***	2.31***	2.94***	2.85***	3.92***	2.65**	3.57***
health	[2.13,3.13]	[2.76,4.06]	[1.70,3.14]	[2.19,3.94]	[2.18,3.73]	[2.96,5.18]	[1.31,5.39]	[1.81,7.06]
Underlying	0.99	1.17	0.89	0.95	0.99	1.26	1.04	1.24
health condition	[0.83,1.17]	[0.98,1.38]	[0.65,1.22]	[0.70,1.29]	[0.80,1.24]	[0.99,1.60]	[0.63,1.72]	[0.77,2.00]
Worked ≥ 40 hours	1.44***	1.27**	1.32	1.25	1.55***	1.25	1.12	1.27
	[1.21,1.70]	[1.07,1.52]	[0.95,1.82]	[0.92,1.70]	[1.25,1.93]	[0.99,1.59]	[0.66,1.90]	[0.76,2.13]
Smokes	1.04	1.50***	0.8	1.19	1.08	1.58**	2.06	2.44
	[0.83,1.30]	[1.20,1.87]	[0.51,1.25]	[0.80,1.78]	[0.82,1.42]	[1.19,2.11]	[0.79,5.35]	[0.98,6.10]
Had flu vaccine	0.98	1.16	1.03	0.99	0.87	1.27*	1.56	1.37
this season	[0.84,1.15]	[0.98,1.36]	[0.77,1.39]	[0.75,1.31]	[0.71,1.08]	[1.01,1.61]	[0.95,2.57]	[0.86,2.19]
**Household composition**								
Lives w/ child under 5	1.32*	1.03	1.4	1.1	1.29	1.14	1.22	0.62
	[1.06,1.63]	[0.82,1.29]	[0.96,2.06]	[0.75,1.62]	[0.96,1.72]	[0.83,1.58]	[0.65,2.29]	[0.31,1.24]
Lives w/ someone over 60	1.04	0.94	1.59*	1.32	0.91	0.79	0.69	0.89
	[0.86,1.25]	[0.77,1.15]	[1.09,2.33]	[0.91,1.92]	[0.73,1.15]	[0.61,1.02]	[0.28,1.69]	[0.39,2.01]
Mean dependent variable	0.20	0.19	0.15	0.17	0.26	0.21	0.14	0.16
Observations	4,993	4,993	1,737	1,737	2,444	2,447	806	806

Notes: Anxiety=1 if Generalized Anxiety Disorder Assessment (GAD-7) ≥ 10, =0 otherwise; Depression=1 if Patient Health Questionnaire-9 (PHQ-9) ≥ 10, =0 otherwise. All models include occupational indicators and institutional indicators. * p<0.05 ** p<0.01 *** p<0.001. 95% confidence intervals in square brackets.

Additional Figures

Figure S1: Example email invitation June 2020 (COMB)

Col·legi de Metges
de Barcelona

Benvolgut/da,

El COMB està col·laborant amb un grup d'investigadors de la Universitat de Cambridge, la Universitat d'Exeter i la Universitat de Glasgow al Regne Unit per entendre com els treballadors sanitaris estan afectats per la pandèmia COVID-19.

Has estat seleccionat aleatòriament per formar part de la mostra de col·legiats i t'agrairíem molt que participessis en aquest estudi.

Si tens 10 minuts, si us plau ajuda'ns a omplir la següent enquesta breu i anònima:

Accés a l'enquesta

Pots trobar més informació sobre l'enquesta i l'equip d'investigadors aquí:

Més informació

Les teves respostes anònimes ajudaran a entendre les mesures que cal adoptar per ajudar els treballadors sanitaris davant les conseqüències d'aquesta crisi.

T'agraim per endavant la teva participació en l'enquesta,

Dr. Gustavo Tolchinsky
Secretari de la Junta de Govern

Figure S2: Example email invitation June 2020 (RCPSG)

Dear Dr [redacted],

- Sent on behalf of Professor Jackie Taylor, President -

The Royal College of Physicians and Surgeons of Glasgow is collaborating with a group of university researchers at the University of Glasgow, the University of Cambridge and the University of Exeter to understand how healthcare workers are being affected by the COVID-19 pandemic.

If you have 10 minutes to spare, please help us fill in the following short and anonymised survey by visiting the following link:

https://uebs.eu.qualtrics.com/jfe/form/SV_2ohMr8isdmO6AbX

More information about the survey and the research team conducting this survey can be found here:

https://sites.google.com/view/hcws

We thank you in advance for your contribution to this research project by participating in the survey.

Your anonymised responses will help improve our understanding of what measures should be taken to help healthcare workers adjust to the consequences of this crisis.

Many thanks,

David Thomson

David Thomson
Global Engagement Officer, Membership and Global Engagement Unit
Royal College of Physicians and Surgeons of Glasgow
232 - 242 St Vincent Street, Glasgow, G2 5RJ
T + 44 (0)141 221 6072 | F + 44 (0)141 221 1804

Figure S3: Example email invitation November 2020 (RCSEd)

THE ROYAL COLLEGE OF SURGEONS OF EDINBURGH

HCWS UNIVERSITY OF CAMBRIDGE UNIVERSITY OF EXETER University of Glasgow

The Effects of a Second Wave on Healthcare Workers

In June our Members were invited to participate in a study conducted by a group of researchers at the University of Glasgow, the University of Cambridge and the University of Exeter to understand how healthcare workers are being affected by the COVID-19 pandemic.

In order to compare the effects of the first and second wave, they would like to invite our Members to participate in a second survey. The survey will help understand what measures should be taken to help healthcare workers adjust to the consequences of this crisis.

If you have 10 minutes to spare, can you please fill in the following short and anonymised survey.

More information about the survey and the research team conducting this survey can be found here.

We thank you in advance for your contribution to this research project by participating in the survey.

START SURVEY

Figure S4: Prevalence of anxiety and depression symptoms by intensity

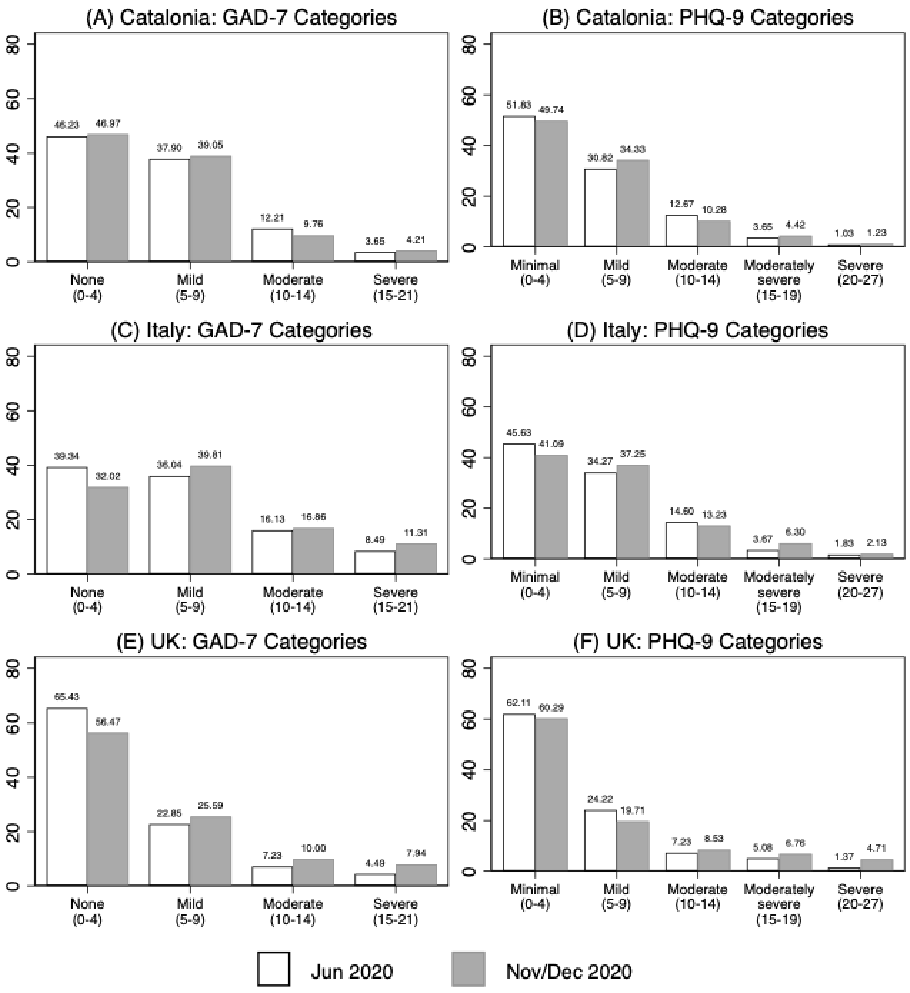

Supplement: S1 Appendix — (PDF) [file pone.0259213.s001.pdf]
